# Nobiletin induces growth inhibition and apoptosis in human nasopharyngeal carcinoma C666‐1 cells through regulating PARP‐2/SIRT1/AMPK signaling pathway

**DOI:** 10.1002/fsn3.953

**Published:** 2019-02-10

**Authors:** Guo Dong Zheng, Ping Jun Hu, Ying Xin Chao, Ying Zhou, Xiu Juan Yang, Bai Zhong Chen, Xi Yong Yu, Yi Cai

**Affiliations:** ^1^ Key Laboratory of Molecular Target & Clinical Pharmacology State Key Laboratory of Respiratory Disease, School of Pharmaceutical Sciences & the Fifth Affiliated Hospital Guangzhou Medical University Guangzhou 511436 China; ^2^ Guangdong Xinbaotang Biological Technology Co, Ltd Jiangmen China

**Keywords:** AMPK, apoptosis, C666‐1, nobiletin, PARP‐2

## Abstract

**Background/Aim:**

Nobiletin, a major polymethoxyflavones (PMFs) from citri reticulatae pericarpium (CRP), can inhibit several forms of cancer proliferation. However, the effects of nobiletin on nasopharyngeal carcinoma (NPC) C666‐1 cells remain largely unknown.

**Materials and Methods:**

Cell counting kit 8 (CCK8) assay was used to measure cell vitality. Flow cytometry was performed to measure the apoptosis rate. Quantitative real‐time polymerase chain reaction (qRT‐PCR) and Western blot analysis were applied to determine the expression of mRNA and protein, respectively.

**Results:**

We showed that the proliferation rate of C666‐1 cells was inhibited and the apoptosis rate was raised after treating with nobiletin. Moreover, nobiletin inhibited the expression of poly(ADP‐ribose)polymerase‐2 (PARP‐2), and the tumor suppression effect of nobiletin on C666‐1 is associated with PARP‐2‐dependent pathway.

**Conclusion:**

We demonstrated for the first time that nobiletin inhibited the growth of C666‐1 cells, which may be relative to its regulation on PARP‐2/SIRT1/AMPK signaling pathway. Our result implied that nobiletin may serve as a strategy to treat nasopharyngeal carcinoma.

## 1 INTRODUCTION

Nasopharyngeal carcinoma (NPC), a distinctive vicious tumor derives from the epithelium of nasopharynx, is the cancer of the highest prevalence in South‐East Asia, especially in southern China (Chen et al., 2010; Kwok et al., 2014). NPC is closely related to Epstein‐Barr virus, which has been regarded as one of the major etiologic factors of NPC (Xiao et al., 2016). In its early stages, NPC is likely to be undetected because it is usually asymptomatic or only presents with clinically insignificant symptoms, which leads to late detection and therapy (Voon et al., 2015). Although radiotherapy and/or chemotherapy have been shown to be effective therapeutic methods for NPC and are able to improve the survival rate, adverse effects and recurrence were also not uncommon (Chan et al., 2015).

Nasopharyngeal carcinoma (NPC), a distinctive vicious tumor derives from the epithelium of nasopharynx, is the cancer of the highest prevalence in South‐East Asia, especially in southern China (Chen et al., 2010; Kwok et al., 2014). NPC is closely related to Epstein‐Barr virus, which has been regarded as one of the major etiologic factors of NPC (Xiao et al., 2016). In its early stages, NPC is likely to be undetected because it is usually asymptomatic or only presents with clinically insignificant symptoms, which leads to late detection and therapy (Voon et al., 2015). Although radiotherapy and/or chemotherapy have been shown to be effective therapeutic methods for NPC and are able to improve the survival rate, adverse effects and recurrence were also not uncommon (Chan et al., 2015).

Nasopharyngeal carcinoma (NPC), a distinctive vicious tumor derives from the epithelium of nasopharynx, is the cancer of the highest prevalence in South‐East Asia, especially in southern China (Chen et al., 2010; Kwok et al., 2014). NPC is closely related to Epstein‐Barr virus, which has been regarded as one of the major etiologic factors of NPC (Xiao et al., 2016). In its early stages, NPC is likely to be undetected because it is usually asymptomatic or only presents with clinically insignificant symptoms, which leads to late detection and therapy (Voon et al., 2015). Although radiotherapy and/or chemotherapy have been shown to be effective therapeutic methods for NPC and are able to improve the survival rate, adverse effects and recurrence were also not uncommon (Chan et al., 2015).

Poly(ADP‐ribose)polymerases (PARPs) are a family of enzymes that catalyze poly(ADP‐ribosyl)ation (PARylation) conserving widespread and highly post‐translational modification (Ali, Khan, Galindo‐Campos, & Yélamos, 2016). Among the 18 members identified so far, poly(ADP‐ribose)polymerase‐1 (PARP‐1) and poly(ADP‐ribose)polymerase‐2 (PARP‐2) are the only proteins stimulated by DNA strand breaks and implicated in the repair of DNA injury (Mégnin‐Chanet, Bollet, & Hall, 2010). Sirtuin 1 (SIRT1), a NAD^+^‐dependent histone deacetylase, is the important downstream target of PARP (Vida, Márton, Mikó, & Bai, 2017). During the last decades, PARP/SIRT1 signaling pathway was extensively studied in metabolic disorders and evidence has suggested its implication in cancer cell biology (Mégnin‐Chanet et al., 2010). Moreover, PARP‐1 inhibition has been reported to reduce the proliferation and promote radiation sensitization in CNE‐2 human NPC cells, which suggested that PARP may be an attractive target for the cancer therapy including NPC (Chen, Zhao, et al., 2015).

**Figure 1:**
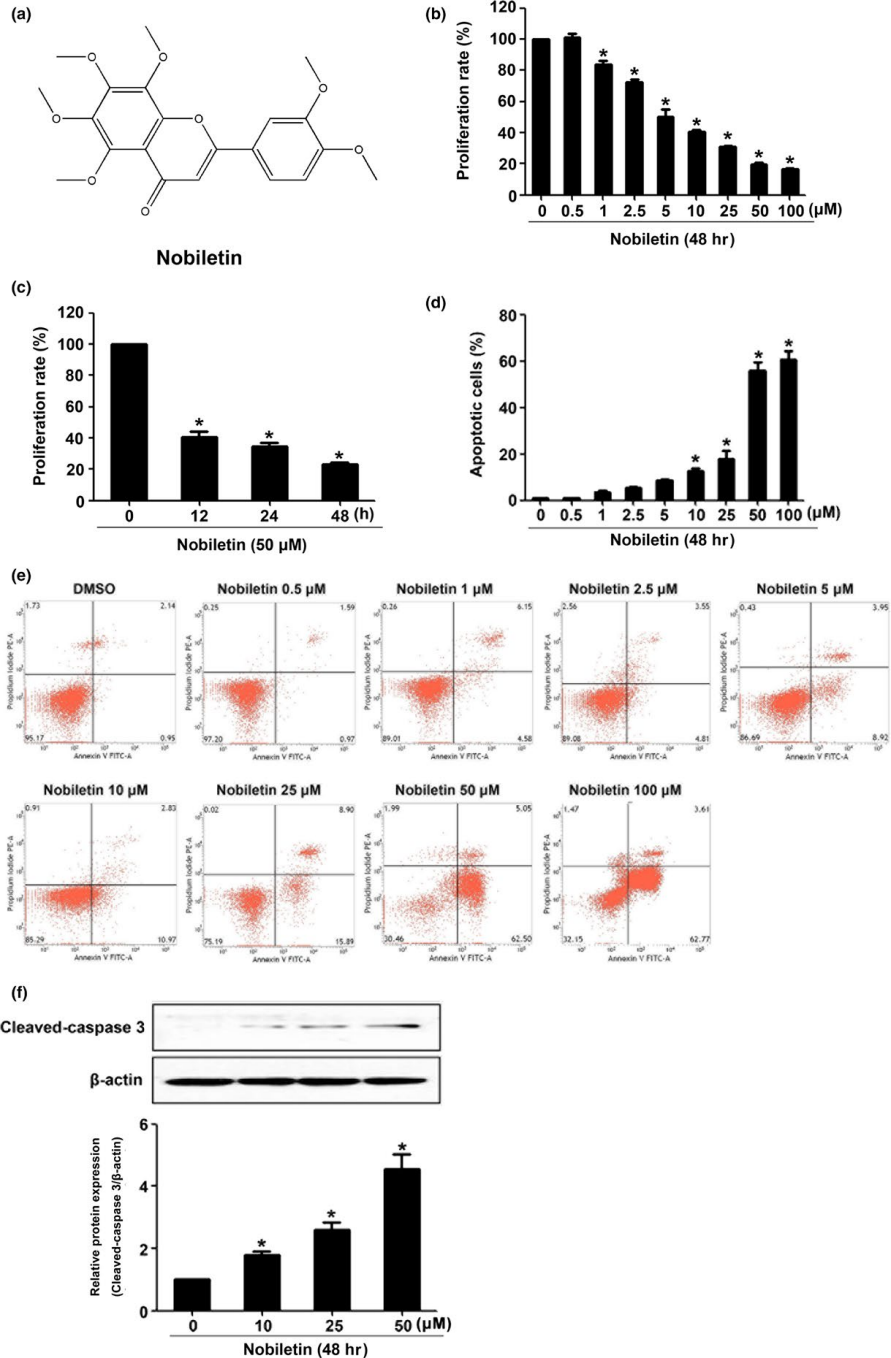
Nobiletin inhibited the cell viability and induced apoptosis in C666‐1 cells. The structure of nobiletin is shown in (A). C666‐1 cells were treated with gradient concentrations of nobiletin for 48 hr or with 50 μM nobiletin for indicated time periods. The cell proliferation rate was detected using CCK8 assay (B and C). The apoptosis rate was detected using flow cytometry (D and E). The expression of cleaved‐caspase 3 was measured with Western blot (F). *p <* *0.05 vs. the group without treatment, *n *=* *3

Many natural products are the potential antitumor drugs because of its low toxicity and few side effects (Mohamed, Jantan, & Haque, 2017; Park et al., 2017). Citri reticulatae pericarpium (CRP), which is prepared from sundried citrus peel, possesses the functions of anti‐inflammation, anticancer, and cardiovascular protection as a traditional Chinese medicine (Fu et al., 2017; Yi, Ma, & Ren, 2017). Apart from essential oil, flavonoids are another primary biological active constituents of CRP and are categorized into flavonoid glycosides and polymethoxyflavones (PMFs) (Luo et al., 2018; Zheng et al., 2013). PMFs consist of nobiletin, tangeretin, 3,5,6,7,8,3′,4′‐heptamethoxyflavone, 5‐hydroxy‐6,7,8,3′,4′‐pentamethoxyflavone (Luo et al., 2018). Nobiletin (Figure 1A), with the highest abundance in PMFs, has attracted extensive attention due to its bioactive effects. Through the last decade, multiple antidisease effects of nobiletin have been discovered, especially its antitumor capability. Nobiletin can be used to treat glioblastoma due to its ability to inhibit the proliferation and migration of glioma cells (Lien et al., 2016). It can also be used as prevention for triple‐negative breast cancer since it can blockade the cell cycle at G0/G1 phase and induce cell apoptosis (Chen, Ono, Takeshima, & Nakano, 2014). Moreover, nobiletin‐induced apoptosis in SNU‐16 cells is regulated via intracellular endoplasmic reticulum stress‐mediated protective autophagy (Moon & Cho, 2016). It had been reported that nobiletin can inhibit the invasion and migration of HONE‐1 and NPC‐BM, the human NPC cell lines (Chien, Hsieh, Chen, Yang, & Chen, 2015). Other than the rest of NPC‐derived cell lines, C666‐1 is the exclusive one that still retains the natural EBV. Thus, it has become an important and representative tool to evaluate the antitumor activity of NPC (Chan et al., 2015). Nevertheless, the effect of nobiletin on human NPC C666‐1 cells and its molecular mechanism are still unclear. The aim of this study is to determine whether nobiletin induces C666‐1 cell apoptosis by regulating PARP‐dependent signaling pathways.

## 2 MATERIALS AND METHODS

### 2.1 Materials and reagents

Dulbecco's modified Eagle's medium (DMEM) was purchased from Gibco (Logan, UT, USA). Fetal bovine serum (FBS) was purchased from Yeasen Biotechnology Co., Ltd. Cell counting kit 8 (CCK8) was purchased from DOJINDO (Japan). Antibodies against cleaved‐caspase 3, p‐AMPK, p‐S6, p‐P70S6K, and SIRT1 were purchased from Cell Signaling Technology (USA); anti‐AMPK and β‐actin were purchased from Santa Cruz Biotech (USA). Antibody against PARP‐2 was purchased from Abcam (UK). Nobiletin was purchased from Chengdu Must Bio‐Technology Co., Ltd (Sichuan, China) and dissolved in dimethyl sulfoxide and stored at −20°C until diluted upon use. Recombinant adenovirus vectors expressing green fluorescent protein (Ad‐Flag) and Flag‐tagged PARP‐2 (Ad‐PARP‐2) were purchased from Genechem Co., Ltd. (Shanghai, China). The viruses were extended in HEK293A cells and purified by virus purification kit (Biomiga, USA), then dialyzed in dilution buffer and stored at −80°C.

### 2.2 Cell culture

C666‐1 cells were cultured in DMEM supplemented with appropriate proportion of FBS at 37°C in humid incubator contains 95% air and 5% CO_2_. Medium was changed every other day. Cells were digested with trypsin (0.25%) and then subcultured when reached 80%–90% confluence. Before treated with nobiletin, cells were cultured in FBS free for 12 hr.

C666‐1 cells were cultured in DMEM supplemented with appropriate proportion of FBS at 37°C in humid incubator contains 95% air and 5% CO_2_. Medium was changed every other day. Cells were digested with trypsin (0.25%) and then subcultured when reached 80%–90% confluence. Before treated with nobiletin, cells were cultured in FBS free for 12 hr.

### 2.3 Cell viability assay

CCK8 was used to detect the cell viability with method described in previous study (Cai, Hong, Zhao, Yue‐Peng, & Qin, 2015). C666‐1 cells were seeded in 96‐well plates with a density of 1 × 10^4^ per well, and circumjacent wells were filled with aseptic phosphate‐buffered saline (PBS). After being cultured in complete medium for 24 hr, the cells changed to FBS‐free medium for another 12 hr at 37°C. Then, cells were treated with nobiletin at the indicated concentration (0, 0.5, 1, 2.5, 5, 10, 25, 50, and 100 μM) or with 50 μM after overexpression of PARP‐2 for 48 hr. CCK8 solution was added 10 μl per well and incubated for 1 hr, followed by reading the absorbance at 450 nm using a Multi‐Volume Spectrophotometer System (BioTek Instruments, Inc., USA).

### 2.4 Flow cytometry

To observe the cell apoptosis, an Annexin V‐FITC Apoptosis Detection Kit (BioVision, USA) and flow cytometry were applied simultaneously with the same protocol as of the previous research (Cai, Zhao, Qin, & He, 2015). Briefly, after 5 × 10^5^ cells reached about 60% confluence, the cells were treated with different concentrations of nobiletin (0, 0.5, 1, 2.5, 5, 10, 25, 50, 100 μM) or treated with 50 μM after overexpression of PARP‐2 for 48 hr. Then, both suspending and adherent cells were collected and resuspended in binding buffer. After the addition of Annexin V‐FITC and PI, the mixture was incubated in the dark at room temperature for 5 min and analyzed immediately with flow cytometer (BD FACSVerse™) and BD FACSuite software.

### 2.5 Western blot analysis

As described previously (Cai, Zhao, Qin, Zhang, & He, 2015), C666‐1 cells (5 × 10^5^ per dish) were seeded into 60‐mm dishes. After incubating for 24 hr, cells were treated nobiletin in different concentrations (0, 10, 25, 50 μM) or treated with 50 μM after overexpression of PARP‐2 for 48 hr. Cells were harvested with detergent‐containing lysis buffer, and protein was separated after 30 min of incubation on ice. Protein concentration was determined with BCA Protein Assay Kit (Thermo Fisher Scientific). Same amount of protein (20 μg per lane) was separated using 12% SDS‐PAGE gel and then transferred to PVDF membrane. The membrane was blocked with 5% nonfat milk in 1× PBST (maybe TBST) for 1 hr at room temperature, then incubated with primary antibodies overnight at 4°C. All primary antibodies were diluted into 1:1,000 upon use. Next, membranes were incubated in anti‐mouse/rabbit secondary antibodies for 1 hr at room temperature. At last, the blotted membranes were visualized by enhanced chemiluminescent (ECL) method and films were then developed.

### 2.6 Quantitative real‐time polymerase chain reaction

Total RNA from C666‐1 cells was extracted with TRIzol, and PrimeScript RT Reagent Kit (Takara Bio Inc., Japan) was used for reverse transcription. PCR primers were designed using the sequences shown in Table 1. mRNA concentrations were measured using Quantitative PCR Kit (Takara Biotechnology) by iCycler iQ system (Bio‐Rad). GAPDH was used as endogenous control. All PCRs were performed in triplicate.

**Table 1:** Primer sequences for qRT‐PCR

Primer	Sequences
PARP‐1	Forward: 5′‐CTAAAGGCTCAGAACGACC‐3′ Reverse: 5′‐GAAGGAGGGCACCGAACA‐3′
PARP‐2	Forward: 5′‐ACAGTGGCACAAATCAAG‐3′ Reverse: 5′‐TACGGAGTCCAAAGTCAT‐3′
SIRT1	Forward: 5′‐CTTGTACGACGAAGACGA‐3′ Reverse: 5′‐TCACCGAACAGAAGGTTAT‐3′
GAPDH	Forward: 5′‐AGGAGTAAGAAACCCTGGAC‐3′ Reverse: 5′‐CTGGGATGGAATTGTGAG‐3′

### 2.7 Statistical analysis

Each result was repeated at least for three times. All data were expressed as means ± *SD*. Unpaired Student's *t* test was performed for the statistical analyses between two groups. The analyses among different groups were carried out with one‐way analysis of variance (ANOVA). In general, statistically significant was defined as *p* < 0.05.

## 3 RESULTS

### 3.1 Nobiletin suppressed the proliferation of C666‐1 cells

The cytotoxicity of nobiletin to C666‐1 cells was detected. C666‐1 cells were treated with nobiletin of gradient concentrations (0, 0.5, 1, 2.5, 5, 10, 25, 50, or 100 μM) for 48 hr, or 50 μM nobiletin was used to treat C666‐1 cells for different time periods (0, 12, 24, or 48 hr), then cell viability was evaluated with CCK8 assay. As shown in Figure 1B,C, the number of survival cells decreased in a dose‐ and time‐dependent manner.

### 3.2 Nobiletin‐induced apoptosis in C666‐1 cells

To clarify whether nobiletin affects cell apoptosis, changes in C666‐1 cells were observed through flow cytometry analysis. After being treated with different doses of nobiletin (0, 0.5, 1, 2.5, 5, 10, 25, 50, 100 μM), the apoptosis rates of C666‐1 cells were detected with flow cytometry. As shown in Figure 1D,E, the percentage of remaining C666‐1 cells was obviously decreased as the concentration of nobiletin increased. Therefore, we concluded that nobiletin induces the apoptosis in C666‐1 cells in a dose‐dependent manner. In addition, cleaved‐caspase 3 was also decreased by nobiletin treatment in a dose‐dependent manner (Figure 1F).

### 3.3 Nobiletin inhibited the expression of PARP‐2

**Figure 2:**
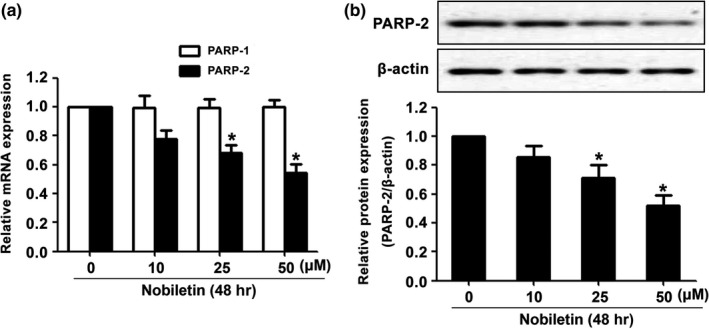
The expression of PARP‐1 and PARP‐2 mRNA and the expression of PARP‐2 protein after being treated with different concentrations of nobiletin. C666‐1 cells were treated with increasing concentrations of nobiletin for 48 hr. The expression of PARP‐1 and PARP‐2 mRNA was measured by qRT‐PCR (A). The protein expression of PARP‐2 protein was detected by Western blot (B). β‐Actin was used as control. *p <* *0.05 vs. the group without treatment, *n *=* *3

As shown in Figure 1, the nobiletin‐induced apoptosis had obvious variation, while the dose was larger than 10 μM. We also noticed that the effect of nobiletin in 50 and 100 μM is indistinctive. Thus, 0, 10, 25, and 50 μM were selected to perform the following experiments. The results in Figure 2A indicated that mRNA expression of PARP‐2 was decreased after being treated with nobiletin in gradient concentration while the mRNA expression of PARP‐1 remained the same. In addition, as shown in Figure 2B, the expression of PARP‐2 was downregulated by nobiletin in a dose‐dependent manner.

As shown in Figure 1, the nobiletin‐induced apoptosis had obvious variation, while the dose was larger than 10 μM. We also noticed that the effect of nobiletin in 50 and 100 μM is indistinctive. Thus, 0, 10, 25, and 50 μM were selected to perform the following experiments. The results in Figure 2A indicated that mRNA expression of PARP‐2 was decreased after being treated with nobiletin in gradient concentration while the mRNA expression of PARP‐1 remained the same. In addition, as shown in Figure 2B, the expression of PARP‐2 was downregulated by nobiletin in a dose‐dependent manner.

### 3.4 PARP‐2 overexpression attenuated the nobiletin‐induced apoptosis

**Figure 3:**
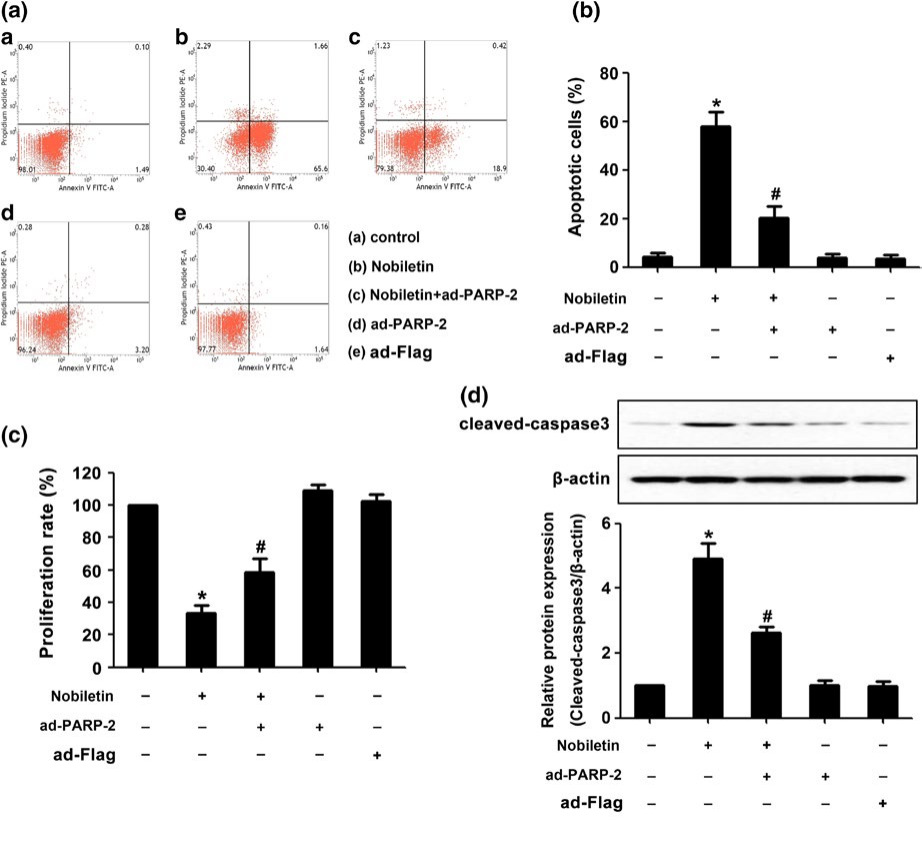
The overexpression of PARP‐2 attenuated the effect of nobiletin on C666‐1 cells. Before being treated with 50 μM nobiletin, C666‐1 cells have been pretreated with PARP‐2 for 1 hr. The groups treated with nobiletin or PARP‐2 only are the positive controls, and the group treated with DMSO is the negative control. After being treated for 48 hr, the cells were applied to perform flow cytometry (A–C) or protein extraction for Western blot of cleaved‐caspase 3 and β‐actin (D). *p <* *0.05 vs. the group without treatment, *n *=* *3. ^#^ *p *<* *0.05 vs. the group treating with nobiletin, *n *=* *3

To explore the function of PARP‐2 in nobiletin‐induced apoptosis, C666‐1 cells were transfected with PARP‐2. Overexpression of PARP‐2 significantly increased the mRNA and protein level of PARP‐2 (Supporting Information Figure S1A,B). Next, we measured the apoptosis rate and apoptosis‐related protein expression. As shown in Figure 3A,B, nobiletin treatment led to significant increase in cell apoptosis, which could be attenuated by preincubation with PARP‐2 overexpression for 24 hr and subsequently treated with nobiletin for 24 hr. Moreover, PARP‐2 overexpression also increased the proliferation rate of C666‐1, which was inhibited by nobiletin (Figure 3C). Western blotting analysis also showed that PARP‐2 could inhibit the protein level of cleaved‐caspase 3 induced by nobiletin (Figure 3D).

### 3.5 PARP‐2 inhibited expression of SIRT1 induced by nobiletin

**Figure 4:**
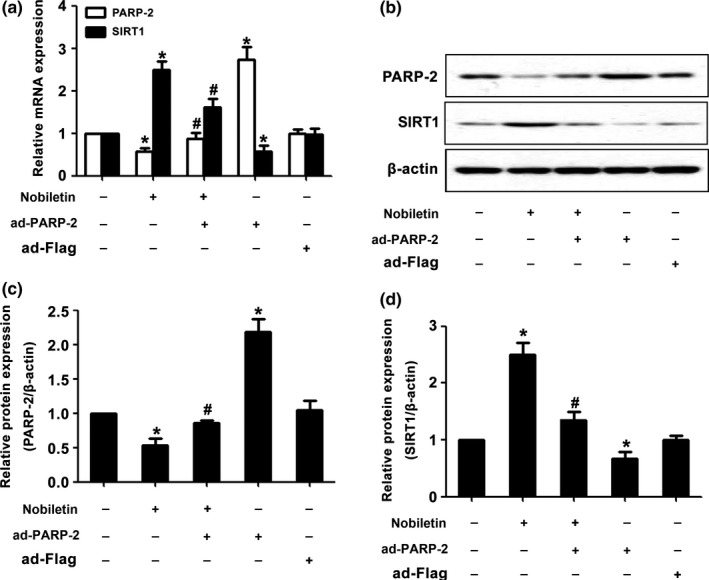
PARP‐2 overexpression reduced the level of SIRT1 induced by nobiletin. Before being treated with 50 μM nobiletin, C666‐1 cells have been pretreated with PARP‐2 for 1 hr. The groups treated with nobiletin or PARP‐2 only are the positive controls, and the group treated with DMSO is the negative control. After being treated for 48 hr, mRNA and protein were extracted to measure the expression of PARP‐2 and SIRT1 (A–D). *p <* *0.05 vs. the group without treatment, *n *=* *3. ^#^ *p* <* *0.05 vs. the group treating with nobiletin, *n *=* *3

The mechanism of how PARP‐2 inhibits nobiletin‐induced apoptosis is unclear. SIRT1 is the important downstream target of PARP. So, we examined the mRNA and protein level of SIRT1. As shown in Figure 4A–D, PARP‐2 overexpression could inhibit the mRNA and protein expression of SIRT1 induced by nobiletin.

### 3.6 Nobiletin‐activated SIRT1/AMPK signaling pathways

**Figure 5:**
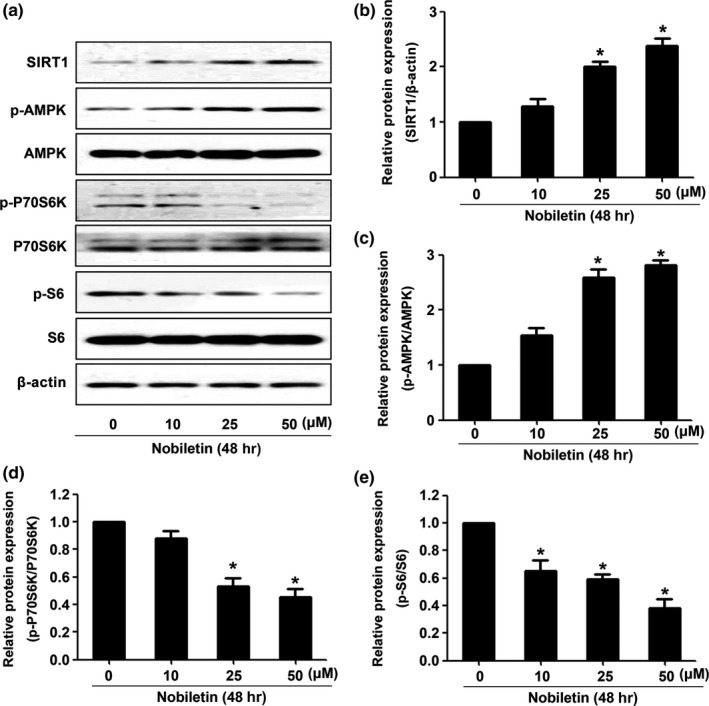
Nobiletin regulated SIRT1/AMPK/mTOR signaling pathways. After being treated with different concentrations of nobiletin, protein was extracted to examine the expression of SIRT1, p‐AMPK, AMPK, p‐P70S6K, P70S6K, p‐S6, and S6 (A–E). *p <* *0.05 vs. the group without treatment, *n *=* *3

We then detected the expression of SIRT1 and its downstream AMP‐activated protein kinase (AMPK)‐dependent signaling pathways. As shown in Figure 5A–E, the SIRT1 expression level and the phosphorylation of AMPK (p‐AMPK) were upregulated. Moreover, the expression of p‐p70S6K and p‐S6 was both downregulated along with AMPK.

## 4 DISCUSSION

In recent years, it has been discovered that nobiletin could inhibit the adhesion, epithelial–mesenchymal transition, metastasis, and invasion of lung cancer (Da, Liu, Zhan, Liu, & Wang, 2016). Moreover, the effect of nobiletin on phosphorylated Akt is the potential method to selectively inhibit ovarian cancer cell proliferation (Chen, Chen, et al., 2015). However, there is little evidence to support the use of nobiletin against NPC. In the present study, we focused on the antiproliferative effect of nobiletin on C666‐1 cells and our results indicated that nobiletin significantly inhibited the viability of NPC C666‐1 cells. Apoptosis, as a programmed cell death, has been known to play a crucial role in obliterating the abnormally proliferated cells (Fan, Yang, & Bi, 2015). Caspase‐3, an enzyme that could cleave most of the caspase substrates in apoptosis pathway, is essential for apoptosis. Activated caspase 3 was regarded as the pivotal slayer to induce cell death in apoptosis (Wu et al., 2016). In the present study, the upregulation of cleaved‐caspase 3 indicated that nobiletin could induce apoptosis in C666‐1 cells. This is also consistent with the result of flow cytometry.

The mechanism why nobiletin mediates its anticancer effects in C666‐1 cells remains unclear. PARPs catalyze a reaction in which the ADP‐ribose moiety of NAD^+^ is transferred to a receptor amino acid, building poly(ADP‐ribose) (PAR) polymers (Mégnin‐Chanet et al., 2010). PARP‐1, the founding member of the PARP superfamily, has been demonstrated to regulate the growth of various tumor cells, such as breast cancer, ovarian cancer, and NPC (Chen, Zhao, et al., 2015; Franzese et al., 2019; Nur Husna, Tan, Mohamud, Dyhl‐Polk, & Wong, 2018). PARP‐2 possesses a catalytic domain structurally similar to PARP‐1, whereas it is less active than PARP‐1 (Ali et al., 2016). Recently, a growing body of evidence also showed that PARP‐2 inhibition served to resist tumor growth via induction of chromosome mis‐segregation, exacerbation of replication stress, and dysregulation of cancer epigenetics, which suggested that PARPs, especially PARP‐1 and PARP‐2, may be an attractive target for cancer therapy (Mégnin‐Chanet et al., 2010). In this study, we showed that nobiletin inhibited the mRNA and protein level of PARP‐2. Moreover, we found that PARP‐2 overexpression could abolish partly the effect of nobiletin on C666‐1 cells, which suggested PARP‐2 may participate in the growth inhibition and apoptosis induced by nobiletin in C666‐1 cells.

SIRT1, a NAD^+^‐dependent histone deacetylase, is the important downstream target of PARP (Vida et al., 2017). PARP‐1 and PARP‐2 regulate SIRT1 via different mechanisms. PARP‐1 increase SIRT1 activity indirectly through the modulation of NAD^+^ levels (Pinton et al., 2013). Instead, PARP‐2 is found to bind directly to the SIRT1 proximal promoter where it acts to negatively regulate SIRT1 expression (Chung & Joe, 2014). SIRT1 involves in cancer progression already obtaining cumulative evidence, but its exact role in carcinogenesis remains controversial. Studies showed that silencing SIRT1 inhibited cell proliferation and tumor formation in some human cancer cell lines such as non‐small‐cell lung cancer and breast cancer (Abdolvahabi et al., 2018; Xu et al., 2018). But another studies claimed that SIRT1 inhibits tumor progression and invasion in human gastric cancer cell lines (Dong et al., 2018). Thus, it remains controversial whether SIRT1 acts as a tumor promoter or suppressor. In this present study, we found that nobiletin could increase the protein level of SIRT1, which suggested that SIRT1 activation may inhibit the growth of C666‐1 cells.

AMPK, a key regulator of energy metabolism, is one of the substrates of SIRT1 and activated by phosphorylation (Cheng et al., 2018; You, Cheng, Yu, Duan, & Peng, 2018). Besides, AMPK is reported to be related to cell cycle and apoptosis (Shrestha et al., 2016). Moreover, a large number of studies have shown that multiple anticancer agents could activate AMPK‐dependent cell death pathways (An et al., 2014; Gao, Ge, & Sun, 2019). AMPK may induce cancer cell death via regulating multiple downstream signal targets, including in‐activating cancer‐promoting mammalian target of rapamycin (mTOR) signaling (Chen, Zhao, et al., 2015). Recent study indicated that 4‐*O*‐methyl‐ascochlorin induced the human leukemia cells by suppressing c‐Myc protein synthesis via an AMPK/mTOR‐dependent mechanism (Shin et al., 2016). In addition, AMPK knocking down prevents capsaicin‐induced cell death in hepatocellular carcinoma cells (Bort, Spínola, Rodríguez‐Henche, & Díaz‐Laviada, 2017). Our previous study also demonstrated that resveratrol could inhibit the growth of NPC cell line C666‐1 through AMPK activation, which means AMPK could be a therapeutic target in NPC (Cai, Zhao, Qin, Zhang, et al., 2015). Consistent with our previous studies, we also showed that nobiletin could activate AMPK and inhibit mTOR signaling, as manifested by dephosphorylation of P70S6K and S6 in C666‐1 cells.

**Figure 6:**
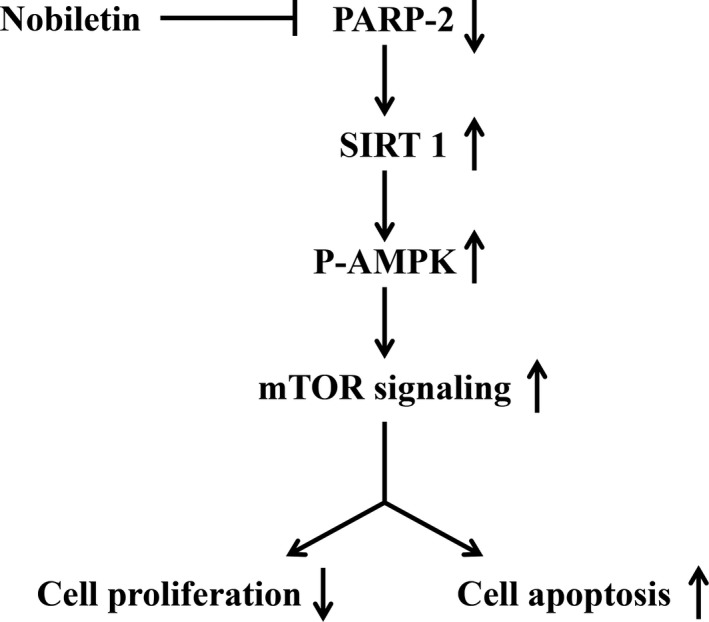
Model for nobiletin inhibiting proliferation and inducing apoptosis. Nobiletin inhibits PARP‐2 level, which activates SIRT1, regulates the downstream AMPK‐mTOR signaling, and suppresses the growth of C666‐1 cell

In conclusion, this study revealed that nobiletin has the ability to significantly inhibit the proliferation of and induce the apoptosis in C666‐1 cells. PARP‐2/SIRT1/AMPK signaling pathway might be the potential molecular mechanism underlying nobiletin‐induced apoptosis (Figure 6). Therefore, we suggest nobiletin as a strategy to treat NPC.

## CONFLICT OF INTEREST

The authors declare no conflict of interest.

## ETHICAL STATEMENT

This study does not involve any human or animal testing.
