# Supplementary material for: Nobiletin induces growth inhibition and apoptosis in human nasopharyngeal carcinoma C666‐1 cells through regulating PARP‐2/SIRT1/AMPK signaling pathway
Source: Food Sci Nutr. 2019 Feb 10;7(3):1104–12. doi: 10.1002/fsn3.953 (PMC6418462; doi:10.1002/fsn3.953)
Supplement: Supplementary file 1 [file FSN3-7-1104-s001.docx]

**Nobiletin induces growth inhibition and apoptosis in human nasopharyngeal carcinoma C666-1 cells through regulating PARP-2/SIRT1/AMPK signaling pathway**

**Supplementary Data**

Supplementary Figure 1


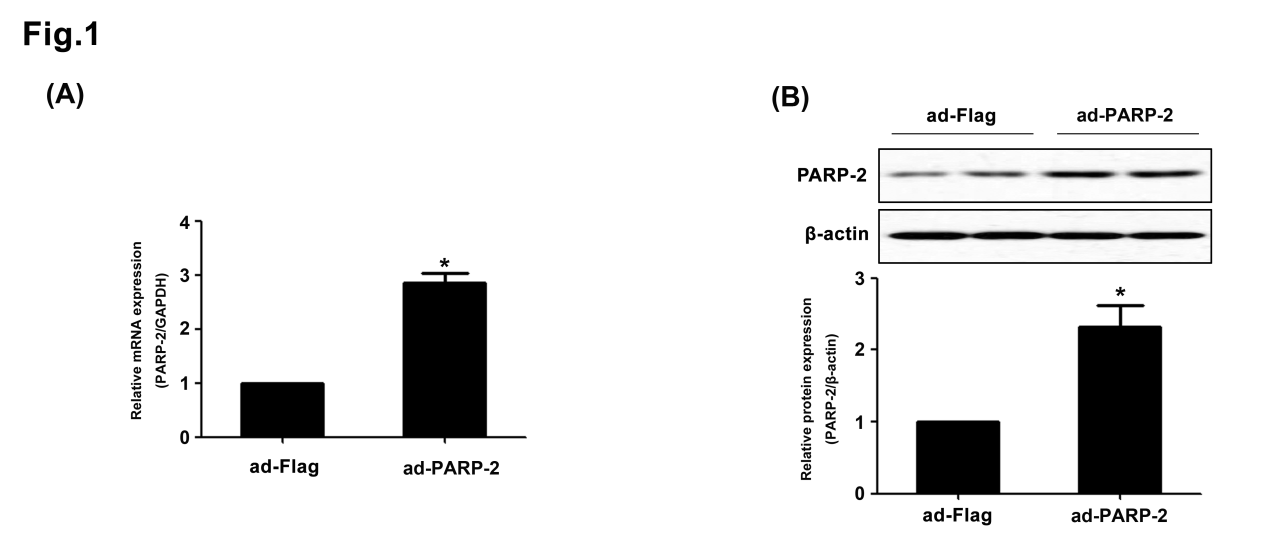


**FIGURE 1** The mRNA and protein expression of PARP-2 after overexpression the PARP-2. Recombinant adenovirus vectors expressing green fluorescent protein (Ad-Flag) and Flag-tagged PARP-2 (Ad-PARP-2) were added in C666-1 cells for 48h, and then the mRNA and protein were extracted to evaluated the expression (A and B).
